# Enhancing Age-Friendly Care with Shared Decision-Making Tools and Technology for Older Adults

**DOI:** 10.1093/geroni/igaf122.499

**Published:** 2025-12-31

**Authors:** Gee Kim

**Affiliations:** FAIR Health, New York, New York, United States

## Abstract

Shared decision-making (SDM) tools powered by technology may improve age-friendly care for older patients with chronic conditions by facilitating SDM with clinicians. Delivered as interactive visual aids on platforms such as iPads or desktop computers, these tools present complex medical and cost information in simplified formats, enhancing patients’ and caregivers’ ability to access and act upon the information. For patients with hip osteoarthritis, for example, digital SDM tools improve communication with healthcare providers, enabling patients to express their preferences and concerns and decide between nonsurgical treatments and surgery. The cost information in the SDM tools allows patients and caregivers to weigh financial implications alongside clinical factors. This session will present results from four clinical sites that have achieved Age-Friendly Health Systems recognition and are collaborating with FAIR Health to use its SDM tools at the point of care, under a grant from The John A. Hartford Foundation. Early feedback suggests that using the tools to facilitate SDM discussions can improve patients’ understanding of treatment options, increase confidence in decision making and foster more productive discussions with providers. Clinicians report that SDM tools help structure conversations, enhance patient comprehension and support collaborative decision making. SDM tools that leverage technology to present treatment information may improve health literacy, reduce decisional conflict and lead to treatment plans that better reflect individual values, goals and financial considerations. Integrating such tools into clinical practice fosters personalized care, improves patient satisfaction and optimizes decision making based on “what matters” to patients, a cornerstone of age-friendly healthcare.

